# Supplementary material for: Multisensory stimuli enhance the effectiveness of equivalence learning in healthy children and adolescents
Source: PLoS One. 2022 Jul 29;17(7):e0271513. doi: 10.1371/journal.pone.0271513 (PMC9337650; doi:10.1371/journal.pone.0271513)
Supplement: Attachment — Submitted filename: Response to Reviewers_2.docx [file pone.0271513.s003.docx]

**First of all, we would like to express our gratitude to both Referees for their help to improve the quality of our study. We discussed all criticisms and suggestions of the Referees and made changes accordingly. The suggestions are answered below point-by-point; textual changes are marked in the final manuscript.**

Reviewer #1: The authors have adequately addressed all review comments. This work makes an interesting contribution to the field of equivalence learning and the role of sensory modalities (visual and multisensory) in various age groups.

A few minor English/grammar corrections to note:

Refer to lines 22, 25, 411, and 525 in the tracked version

**Answer: The English/grammar corrections were performed with the help of a native English speaker.**

Reviewer #2: The authors have largely addressed the points raised in the reviews, and the manuscript has been improved. However, I have one remaining comment:

Authors now report correlations between age and performance in each performance metric individually (i.e., separately for the V and the AV paradigms), and find, maybe not surprisingly, that performance generally increases with age. However, my original comment pertained to the correlation between age and the size of the multisensory gain (i.e., the performance difference between V and AV paradigms, e.g., MNAT minus VNAT). At least for NAT and ALER, the results presented in Tables 3 and 4 seem to suggest that the multisensory gain becomes smaller in >12 as compared to <12, and I wonder if there would be a significant correlation here? If yes, this would indicate that the multisensory gain actually decreases and becomes more adult-like over development, which would be different to the current interpretation that there is no difference until age 18.

Similarly, in Table 5 the authors did not directly test whether there is a significant difference between <12 and >12 in the multisensory gain, they only compare V and AV metrics separately between age groups. I think it would be crucial to directly compare the size of the multisensory gain between age groups (note that multisensory gain could be significant in both age groups as shown in Table 4 but still be significantly smaller in >12 than in <12).

**Answer: We have defined the multisensory gain in Data Analysis section. We have calculated the gain values of each psychophysical learning parameters (NAT, ALER, RER, GER), and made the correlations of these parameters with age. Significant correlation of multisensory gain and age was found in acquisition phase. This indicates that the multisensory gain actually decreases with the aging of the children and becomes more adult-like over development. We have added a new paragraph “*Correlation of multisensory gain with age and the size of multisensory gain in the age groups”* to the results section. Two new tables were prepared (Table 6 and Table 7), which contain the descriptive statistic and the correlation results in detailed.**

**We have also compared the multisensory gains between the two age groups (under and above 12 years). The comparison of the multisensory gains of the two age cohorts denoted that the older cohort has smaller gain than the younger one, and the differences are significant in the acquisition phase. Table 8 contain the detailed results of the Mann-Whitney U tests.**

**We have additionally modified the Abstract and Discussion sections according to these new results.**
